# CDCP1 expression is frequently increased in aggressive urothelial carcinoma and promotes urothelial tumor progression

**DOI:** 10.1038/s41598-022-26579-z

**Published:** 2023-01-02

**Authors:** Miriam Saponaro, Sina Flottmann, Markus Eckstein, Oliver Hommerding, Niklas Klümper, Dillon Corvino, Sana Hosni, Anja Schmidt, Nicolas Mönig, Doris Schmidt, Jörg Ellinger, Marieta Toma, Glen Kristiansen, Tobias Bald, Andrea Alimonti, Manuel Ritter, Michael Hölzel, Abdullah Alajati

**Affiliations:** 1 Department of Urology and Pediatric Urology, University Medical Center Bonn (UKB), Venusberg-Campus 1, 53127 Bonn, Germany; 2 Institute of Pathology, University Hospital Erlangen, Friedrich-Alexander-Universität Erlangen-Nürnberg (FAU), Erlangen, Germany; 3 Institute of Pathology, University Medical Center Bonn (UKB), Bonn, Germany; 4 Institute of Experimental Oncology, University Medical Center Bonn (UKB), Bonn, Germany; 5 Institute of Oncology Research, Università Della Svizzera Italiana, Bellinzona, Switzerland

**Keywords:** Tumour biomarkers, Urological cancer

## Abstract

The prognosis of patients with advanced urothelial carcinoma (UC) remains poor and improving treatment continues to be a major medical need. CUB domain containing protein 1 (CDCP1) is a known oncogene in various types of solid cancers and its overexpression is associated with impaired prognosis. However, its role in UC remains undetermined. Here we assessed the clinical relevance of CDCP1 in two cohorts of UC at different stages of the disease. Immunohistochemistry showed that CDCP1 is highly expressed in advanced UC, which significantly correlates with shorter overall survival. Importantly, the basal/squamous UC subtype showed significantly enriched CDCP1 at the mRNA and protein levels. The functional role of CDCP1 overexpression was assessed taking advantage of ex vivo organoids derived from the CDCP1^pcLSL/+^ transgenic mouse model. Furthermore, CDCP1 knockout UC cell lines were generated using CRISPR/Cas9 technology. Interestingly, CDCP1 overexpression significantly induced the activation of MAPK/ERK pathways in ex vivo organoids and increased their proliferation. Similarly, CDCP1 knockout in UC cell lines reduced their proliferation and migration, concomitant with MAPK/ERK pathway activity reduction. Our results highlight the relevance of CDCP1 in advanced UC and demonstrate its oncogenic role, suggesting that targeting CDCP1 could be a rational therapeutic strategy for the treatment of advanced UC.

## Introduction

The standard treatment for advanced urothelial cancer (UC) is radical cystectomy preceded or followed by platinum-based chemotherapy^1^. The advent of immunotherapy and, more recently, the use of antibody–drug conjugates (ADC) has broadened the therapeutic armamentarium^2^. However, the prognosis of patients with advanced UC remains poor and effective treatment remains a major medical need. CUB domain containing protein 1 (CDCP1), also known as SIMA135^3^, gp140^4^, CD318^5^, or Trask^4^, is a transmembrane protein that is frequently overexpressed in a variety of human cancers^6^. In the cell membrane, CDCP1 exists in two forms as the 135 kDa full-length (FL) protein can be cleaved by serine proteases at arginine-368 and lysine-369. Its proteolysis results in the formation of a soluble 65 kDa fragment and a membrane-spanning fragment of 70 kDa^7^. Both the cleaved (C) and FL forms act as substrates for Src, promoting interactions with several receptor tyrosine kinases (RTKs) thereby representing a key factor for pro-tumoral downstream signaling^7–10^. Accordingly, several studies demonstrated that CDCP1 is a potent oncogene and suggest that its overexpression is functionally involved in disease progression^6, 11–13^. Indeed, elevated levels of this protein are associated with more advanced stages, poorer prognoses, and/or therapy responses in all studied malignancies^11, 12, 14–24^. Our recent study carried out on a novel spontaneous mouse model of prostate cancer showed that CDCP1 promotes progression and metastasis through the upregulation of MAPK/ERK and AKT pathways^12^. Similarly, other studies describe CDCP1 overexpression as a driver of MAPK/ERK- and AKT-dependent tumor progression^8, 11^. Interestingly, CDCP1 targeting, either with monoclonal antibodies or small molecule inhibitors, has demonstrated effectiveness in inhibiting tumor growth and metastasis in vivo^25^. Moreover, a recent study identified CDCP1 as a suitable target for CAR T-cell-based immunotherapy in pancreatic cancer^26^. Since treatments with either SRC or MAPK/ERK inhibitors have been associated with poor tolerability in the clinic^27^, CDCP1 targeting could represent an excellent alternative therapeutic option. However, to the best of our knowledge, the role of CDCP1 in UC has not been well described, and more studies to assess it are needed^28^.

## Results

### UC exhibits an elevated expression of CDCP1, which correlates with shorter overall survival in UC patients

**Figure 1:**
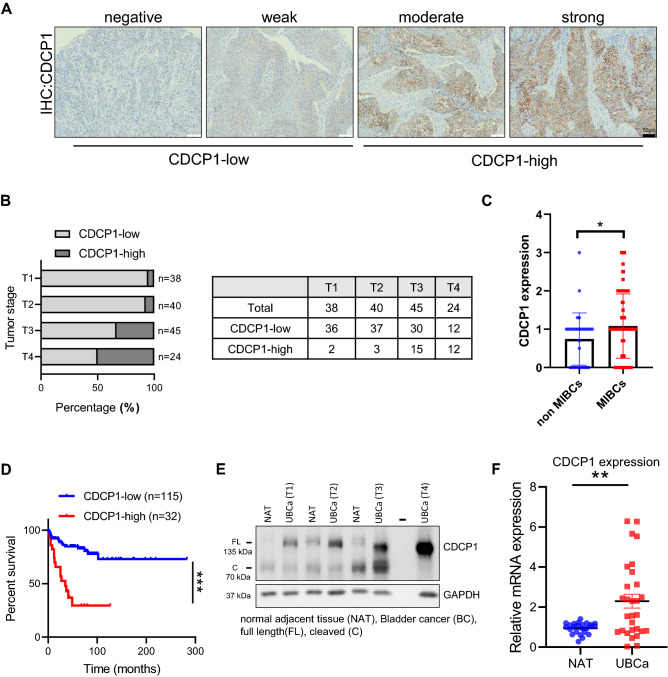
UC exhibits elevated expression of CDCP1, which correlates with shorter overall survival in UC patients. (**A**) Representative images of CDCP1-low and CDCP1-high tumors. (**B**) Percentage of patients expressing low and high CDCP1 levels, divided by the tumor stage (T1, T2, T3, T4). The table indicates the relative number of patients, which express low and high CDCP1 divided per tumor stage. (**C**) Column bar graph comparing the expression of CDCP1 in NMIBC (T1) and MIBC (T2–T4). Error bars indicate SD. *P < 0.05. Statistical test: two-tailed unpaired t test. (**D**) Kaplan–Meier survival analysis of UC patients stratified based on the semi-quantitative expression of CDCP1 (CDCP1-low: negative, weak; CDCP1-high: moderate, strong). ***P < 0.001. Statistical test: log-rank test. (**E**) Western blot analysis of CDCP1 and GAPDH expression on representative tumor samples from patients presenting lesions at different stages (T1, T2, T3 and T4). CDCP1 tumor expression is compared with its expression in the NAT from the same patients. (**F**) Comparison between the expression of CDCP1 in UC samples and NAT at the transcriptional level. Expression was quantified via real-time quantitative PCR, and normalized to GAPDH. Error bars indicate SD. **P < 0.01. Statistical test: two-tailed unpaired t test.

To assess the clinical relevance of CDCP1 in UC, we first performed immunohistochemistry (IHC) staining of human CDCP1 on paraffin-embedded human bladder cancer samples based on the previously described protocol^12^. We examined a cohort of 147 specimens spanning from T1 to T4 stages (Table 1)^29, 30^ and stratified samples based on their membrane staining for CDCP1 into four groups (negative, weak, moderate and strong). All groups with negative or weak staining intensity were classified as CDCP1-low, while moderate and strong groups were classified as CDCP1-high (Fig. 1A). In line with previous results^28, 31^, CDCP1 expression was negatively or weakly expressed in normal urothelium or normal adjacent tissue (NAT) (Supplementary Fig. 1). A large portion of urothelial tumors at T1 and T2 stages were classified as negative/weak for CDCP1 (CDCP1-low), while the 35% of T3 and 50% of T4 showed high expression levels of CDCP1 (CDCP1-high) (Fig. 1B). Of note, levels of CDCP1 in muscular invasive bladder cancer (MIBC) stages (T2-T4) are significantly higher than in the non-invasive stage (T1) (Fig. 1C). Most importantly, patients with CDCP1-high expression showed a significant poorer outcome compared to patients with CDCP1-low expression levels (Log Rank mantel cox test P < 0.0001) (Fig. 1D). In parallel, we performed western blot and real-time PCR (RT-PCR) analyses (n = 10 and n = 107 respectively) on additional UC frozen samples and the corresponding NAT. These analyses demonstrated a robust increase of CDCP1 expression at protein and RNA levels in UC when compared to NAT (Fig. 1E,F). Taken together, these data showed that CDCP1 is overexpressed in UC compared to normal tissues, and its overexpression is associated with advanced UC and shorter overall survival (OS).

**Table 1:** Characterization of cohort 1.

Age		
Average (range)	68 (38; 94)	

Sex	N	% of total
Female	46	25.7%
Male	132	74.3%

Primary tumor (location)	N	% of total
Control	31	19.0%
BCa	136	81.0%

Tumor stage	N	% of total
Control	31	–
min.pT1	38	26.0%
min.pT2	40	27.0%
min.pT3	45	31.0%
min.pT4	24	16.0%

Tumor type	N	% of total
Control	31	–
Non-muscle invasive (NMIBC)	38	26.0%
Muscle invasive (MIBC)	109	74.0%

Survival time (months)		
Median (range)	50 (1; 283)	

CDCP1 level	N	% of total
Low	115	78.3%
High	32	21.7%

### CDCP1 expression is elevated in advanced UC and enriched in Ba/Sq subtype

**Figure 2:**
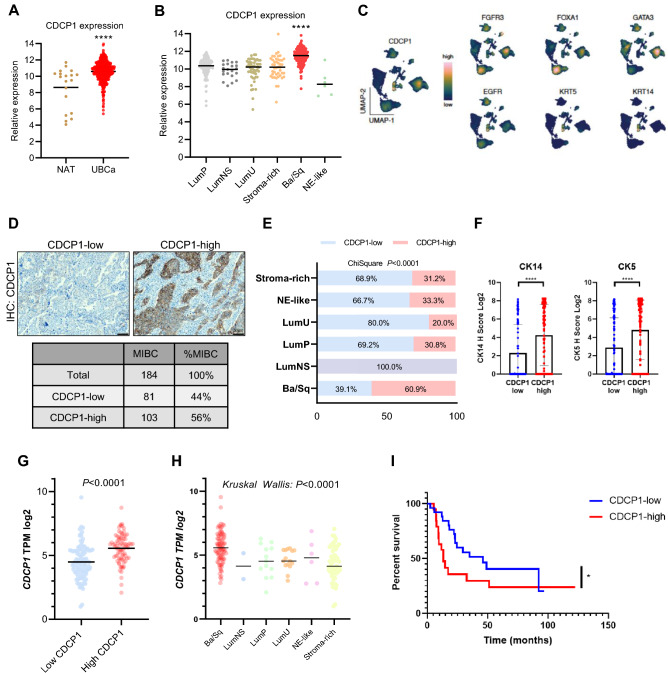
CDCP1 expression is elevated in advanced UC and enriched in Ba/Sq subtype. (**A**) Expressional levels of CDCP1 in UC tissue compared to NAT. Data obtained from the TCGA BLCA data set. ****P < 0.0001. Statistical test: two-tailed unpaired t test. (**B**) Comparison of CDCP1 levels between the UC molecular subtypes from TCGA BLCA data set. ****P < 0.0001. Statistical test: one-way analysis of variance. (**C**) Visualization via scRNAseq analysis of the expressional levels of CDCP1, and different Lu subtype molecular markers (FGFR3, FOXA1, GATA3) and Ba/Sq subtype molecular markers (EGFR, KRT5, KRT14) in the UC cohort from Chen et al., 2020. (**D**) Representative images of tumors from the MIBC TMA showing tumors with CDCP1-low and CDCP1-high. Table showing the total number of MIBC tumors, CDCP1-low and CDCP1-high. (**E**) Percentage of tumors from the MIBC TMA which show a low or high CDCP1 expression, clustered on the base of the UC subtype. (**F**) Bar graphs indicating the expression of CK5 and CK14 in CDCP1-high tumors compared to CDCP1-low tumors. The expression is evaluated by IHC in the MIBC TMA. Error bars indicate SD. ****P < 0.0001. Statistical test: two-tailed unpaired t test. (**G**) Transcriptional expression of CDCP1 (TPM: transcripts per million reads) compared to CDCP1-low and CDCP1-high levels in the MIBC TMA confirming the correlation between CDCP1 transcripts and protein levels. (**H**) CDCP1 TPM across the UC subtypes showing the high expression of CDCP1 in the Ba/Sq subtype. Tumor samples are correspondent to the ones from the MIBC TMA. (**I**) Kaplan–Meier survival analysis of UC patients treated with chemotherapy stratified based on the semi-quantitative expression of CDCP1 (CDCP1-low: negative, weak; CDCP1-high: moderate, strong). *P < 0.05. Statistical test: Gehan–Breslow–Wilcoxon test.

To gain further insights about the expression of CDCP1 in UC, we analyzed CDCP1 expression in two publically available data sets. The first, consisting of 405 tissue specimens of bladder cancer from the cancer genome atlas (TCGA), was classified according to the consensus subtyping approach of muscle-invasive urothelial bladder cancer (MIBC)^32^. In line with our previous results, we observed elevated levels of CDCP1 in UC compared to its NAT (Fig. 2A). Importantly, we found that CDCP1 levels were strongly associated with the Ba/Sq subtype (Fig. 2B). This was confirmed using a second data set, a single-cell-RNA-sequencing (scRNAseq) of human UC samples^33^. Indeed, analysis of this dataset for cells annotated as epithelial origin revealed that expression of CDCP1 and that of markers reported to be enriched in the Ba/Sq subtype, such as EGFR, KRT5 (CK5) and KRT14 (CK14), have a high degree of co-localization (Fig. 2C). Thereafter, we performed IHC of CDCP1 on a well-established prospectively recruited consecutive cohort of MIBC patients treated with radical cystectomy and adjuvant chemotherapy as previously described (Table 2)^32^. The multivariate analysis on this cohort demonstrated that CDCP1 is not an independent variable for the prediction of patients´ prognosis (Table 3). However, out of 184 analyzed tumors 56% were positive for CDCP1 (Fig. 2D) and we found a strong association between CDCP1 protein expression and the Ba/Sq subtype (Fig. 2E). This association with the Ba/Sq subtype of UC was further validated using the Ba/Sq subtype markers CK5 and CK14^32^ (Fig. 2F). Of note, the mRNA levels of CDCP1 reflected the protein levels observed in this cohort (Fig. 2G) and were enriched in the Ba/Sq subtype (Kruskal Wallis: P < 000.1) (Fig. 2H). Together, these results confirm the association of CDCP1 expression with the aggressive Ba/Sq UC subtype. Moreover, considering that several studies have already reported the association of CDCP1 with therapy resistance^6^, we questioned whether this protein has the same impact on MIBC. The OS of chemo-treated patients expressing high CDCP1 levels is clearly reduced (Fig. 2I), suggesting that CDCP1 may be a suitable marker for chemotherapy sensitivity and targeting CDCP1 could serve as a novel therapeutic strategy to treat resistant patients.

**Table 2:** Characterization of cohort 2.

Age (years)		
Average (range)	68,7 (37; 91)	

Sex	N	% of total
Female	46	25.0%
Male	138	75.0%

Primary tumor (location)	N	% of total
BCa	184	100.0%

Grading 2004/2016	N	% of total
High grade	184	100.0%

Grading 1973	N	% of total
G2	5	3.0%
G3	179	97.0%

Initial tumor stage	N	% of total
pT2	53	29.0%
pT3	88	48.0%
pT4	43	23.0%

pN-stage	N	% of total
NX	13	7.0%
pN0	118	64.0%
pN1	19	10.0%
pN2	34	18.0%

Adjuvant chemotherapy	N	% of total
No	139	76.0%
Yes	45	24.0%

Survival time (months)		
Average (range)	37 (0; 153,33)	

CDCP1 level	N	% of total
Low	81	44.0%
High	103	56.0%

Molecular variant	N	% of total
Ba/Sq	87	47.0%
LumNS	2	1.0%
LumP	13	7.0%
LumU	15	8.0%
NE-like	6	3.0%
Stroma-rich	61	33.0%

**Table 3:** Multivariate analysis for CDCP1.

Cox proportional hazards fit					
Censor: disease specific survival censor					
Effect summary					
Source	LogWorth		P value		
pN-stage summary	2.798	+++++++	0.00159		
pT-stage summary	1.64	++++	0.02293		
L	0.908	++	0.12351		
Resection margin	0.888	++	0.12938		
Age	0.782	++	0.1653		
Histology summary	0.771	++	0.16948		
Gender	0.217	+	0.60618		
CDCP1 low vs. high	0.148		0.71175		
V	0.007		0.98386		
WHO 2016 grading	–	–	–		
Whole model					
Number of events	95				
Number of censorings	89				
Total number	184				

AICc	BIC				
858.166	903.533				

Model	−LogLikelihood	ChiSquare	DF	Prob > Chisq	
Difference	34.3554	68.7108	15	0	
Full	412.6543				
Reduced	447.0097				
Parameter estimates					

Term	Estimate	Std error	Lower 95%	Upper 95%	
CDCP1 low vs. high [low]	0.043	0.117	−0.18	0.28	
pT-stage summary [pT2]	−0.55	0.228	−1.02	−0.11	
pT-stage summary [pT3]	0.057	0.153	−0.24	0.36	
pN-stage summary [pN+]	0.047	0.178	−0.3	0.4	
pN-stage summary [pN0]	−0.677	0.187	−1.04	−0.31	
Gender [female]	0.063	0.122	−0.18	0.3	
Age	0.013	0.01	−0.01	0.03	
Resection margin [R1-R0]	0.29	0.281	−0.28	0.82	
Resection margin [RX-R1]	2.301	1.098	−0.66	4.1	
L[1-0]	0.433	0.283	−0.12	1	
V[1-0]	0.005	0.252	−0.5	0.49	
Variant summary [neuroendocrine]	1.258	0.512	0.08	2.15	
Variant summary [urothelial-NOS]	−0.505	0.209	−0.91	−0.08	
Variant summary [urothelial-sarcomatoid/rhabdoid]	−0.022	0.286	-0.61	0.53	
Variant summary [urothelial-squamous]	−0.371	0.253	-0.87	0.12	
Effect Wald tests					

Source	Nparm	DF	Wald ChiSquare	Prob > ChiSq	
CDCP1 low vs. high	1	1	0.14	0.712	
pT-stage summary	2	2	7.49	0.024	
pN-stage summary	2	2	13.24	0.001	
Gender	1	1	0.27	0.604	
Age	1	1	1.89	0.169	
Resection margin	2	2	6.48	0.039	
WHO 2016 grading	0	0	0		
L	1	1	2.34	0.126	
V	1	1	0	0.984	
Variant summary	4	4	8.54	0.074	
Risk ratios					
Unit risk ratios					

Per unit change in regressor					
Term	Risk ratio	Lower 95%	Upper 95%	Reciprocal	
Age	1.01	0.99	1.03	0.9867414	
Range risk ratios					

Per change in regressor over entire range					
Term	Risk ratio	Lower 95%	Upper 95%	Reciprocal	
Age	2.06	0.75	5.86	0.4863856	

Risk ratios for CDCP1 low vs. high					
Level1	/Level2	Risk ratio	Prob > Chisq	Lower 95%	Upper 95%
High	Low	0.92	0.712	0.58	1.45
Low	High	1.09	0.712	0.69	1.74

Risk ratios for pT-stage summary					
Level1	/Level2	Risk ratio	Prob > Chisq	Lower 95%	Upper 95%
pT3	pT2	1.84	0.069	0.96	3.69
pT4	pT2	2.83	0.006	1.34	6.16
pT4	pT3	1.54	0.087	0.94	2.52
pT2	pT3	0.54	0.069	0.27	1.05
pT2	pT4	0.35	0.006	0.16	0.75
pT3	pT4	0.65	0.087	0.4	1.07

Risk ratios for pN-stage summary					
Level1	/Level2	Risk ratio	Prob > Chisq	Lower 95%	Upper 95%
pN0	pN+	0.48	0.009	0.28	0.83
pNX	pN+	1.79	0.131	0.83	3.66
pNX	pN0	3.7	0.002	1.68	7.76
pN+	pN0	2.06	0.009	1.2	3.56
pN+	pNX	0.56	0.131	0.27	1.2
pN0	pNX	0.27	0.002	0.13	0.6

Risk ratios for gender					
Level1	/Level2	Risk ratio	Prob > Chisq	Lower 95%	Upper 95%
Male	female	0.88	0.606	0.55	1.44
Female	Male	1.14	0.606	0.69	1.82

Risk ratios for resection margin					
Level1	/Level2	Risk ratio	Prob > Chisq	Lower 95%	Upper 95%
R1	R0	1.34	0.312	0.75	2.28
RX	R0	13.34	0.074	0.7	76.53
RX	R1	9.98	0.104	0.52	60.06
R0	R1	0.75	0.312	0.44	1.33
R0	RX	0.07	0.074	0.01	1.42
R1	RX	0.1	0.104	0.02	1.93

Risk ratios for L					
Level1	/Level2	Risk ratio	Prob > Chisq	Lower 95%	Upper 95%
1	0	1.54	0.124	0.89	2.71
0	1	0.65	0.124	0.37	1.12

Risk ratios for V					
Level1	/Level2	Risk ratio	Prob>Chisq	Lower 95%	Upper 95%
1	0	1.01	0.984	0.61	1.64
0	1	0.99	0.984	0.61	1.64

Risk ratios for variant summary					
Level1	/Level2	Risk ratio	Prob>Chisq	Lower 95%	Upper 95%
Urothelial-NOS	Neuroendocrine	0.17	0.024	0.05	0.61
Urothelial-sarcomatoid/rhabdoid	Neuroendocrine	0.28	0.1	0.07	1.1
Urothelial-sarcomatoid/rhabdoid	Urothelial-NOS	1.62	0.192	0.8	3.28
Urothelial-squamous	Neuroendocrine	0.2	0.038	0.05	0.73
Urothelial-squamous	Urothelial-NOS	1.14	0.669	0.62	2.11
Urothelial-squamous	Urothelial-sarcomatoid/rhabdoid	0.71	0.385	0.32	1.53
Urothelial-variant histology	Neuroendocrine	0.2	0.038	0.05	0.74
Urothelial-variant histology	Urothelial-NOS	1.16	0.6	0.67	1.98
Urothelial-variant histology	Urothelial-sarcomatoid/rhabdoid	0.71	0.365	0.35	1.46
Urothelial-variant histology	Urothelial-squamous	1.01	0.976	0.52	1.97
Neuroendocrine	Urothelial-NOS	5.83	0.024	1.63	20.81
Neuroendocrine	Urothelial-sarcomatoid/rhabdoid	3.6	0.1	0.91	14.27
Urothelial-NOS	Urothelial-sarcomatoid/rhabdoid	0.62	0.192	0.31	1.25
Neuroendocrine	Urothelial-squamous	5.1	0.038	1.36	19.11
Urothelial-NOS	Urothelial-squamous	0.87	0.669	0.47	1.61
Urothelial-sarcomatoid/rhabdoid	Urothelial-squamous	1.42	0.385	0.65	3.08
Neuroendocrine	Urothelial-variant histology	5.05	0.038	1.36	18.76
Urothelial-NOS	Urothelial-variant histology	0.87	0.6	0.51	1.48
Urothelial-sarcomatoid/rhabdoid	Urothelial-variant histology	1.4	0.365	0.68	2.88
Urothelial-squamous	Urothelial-variant histology	0.99	0.976	0.51	1.93

### Transgenic overexpression of CDCP1 induces proliferation in ex vivo mouse organoids and its knockout inhibits proliferation and migration of UC cells

**Figure 3:**
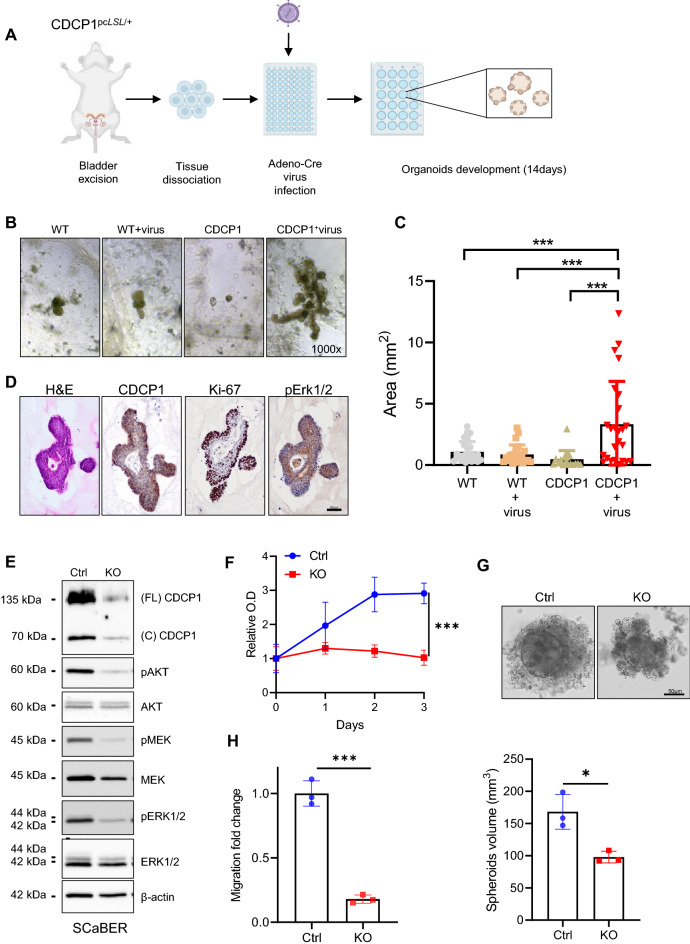
Transgenic overexpression of CDCP1 induces proliferation in ex vivo mouse organoids and its knockout inhibits proliferation and migration of UC cells. (**A**) Schematic representation of the generation of mouse organoids. (**B**) Representative images of the organoids generated from the C57BL/6 (WT) and transgenic (CDCP1) mice after 14 days of culture. The WT+ virus and the CDCP1+ virus conditions are transduced with the adeno-CRE virus. The WT and the CDCP1 conditions are processed as WT+ virus and CDCP1+ virus in the absence of the adeno-CRE virus. WT, WT+ virus and CDCP1 conditions are all controls. (**C**) Quantification of the approximate area of the organoids. Error bars indicate SD. ***P < 0.001. Statistical test: one-way analysis of variance. (**D**) Representative images of H&E and IHC (CDCP1, Ki67, p-ERK1/2) performed on the organoids. (**E**) Western blot analysis of CDCP1 and major downstream targets of CDCP1 signaling in SCaBER UC cell line (Ctrl) and its CDCP1^−/−^ counterpart (KO). (**F**) Relative O.D (proliferation) change of the CDCP1 expressing SCaBER cells (Ctrl) compared to the CDCP1^−/−^ (KO). Error bars indicate SD. ***P < 0.001. Statistical test: two-tailed unpaired t test. (**G**) Representative images of the spheroids originated from the CDCP1+ (Ctrl) and CDCP1^−/−^ (KO) SCaBER cells. Bar graphs show the quantification of the spheres volume. Error bars indicate SD. *P < 0.05. Statistical test: two-tailed unpaired t test. (**H**) Migration fold change of CDCP1 expressing SCaBER (Ctrl) compared to the CDCP1^−/−^ (KO). Error bars indicate SD. ***P < 0.001. Statistical test: two-tailed unpaired t test.

To model the effect of CDCP1 overexpression in UC, we exploited the previously generated transgenic mouse model for CDCP1 (CDCP1^pcLSL/+^) and established an ex vivo 3D organoids system that overexpresses CDCP1^12^. Briefly, the bladder of 8 weeks old male CDCP1^pcLSL/+^ mice was excised and dissociated into single cells, as previously described^33^. Bladder cells were infected with an adeno-Cre virus to induce the expression of CDCP1 and seeded in Matrigel (Fig. 3A). We examined the effect of CDCP1 expression on the growth of bladder mouse organoids over two weeks. CDCP1 overexpression resulted in the formation of larger and morphologically distinctive organoids compared to the controls (Fig. 3B). Quantification of the organoids area revealed a significant increase in their size when CDCP1 is overexpressed compared to the controls (Fig. 3C). At the molecular level, IHC analysis on mouse organoids confirmed the expression of CDCP1 (Fig. 3D). Importantly, CDCP1 overexpressing organoids expressed high levels of Ki67 and pERK (Fig. 3D). To further assess the relevance of these findings in human UC, we aimed to perform CDCP1 knockout (KO) in CDCP1 expressing cells with the CRISPR/Cas9 method. Firstly, we screened several UC cell lines for their CDCP1 expression. Western blot analysis indicated variable expression of CDCP1 among the tested UC cell lines (Supplementary Fig. 2A). Given the fact that CDCP1 expression was associated with Ba/Sq subtype and it is highly expressed in the Ba/Sq SCaBER cells, we generated KO of CDCP1 in this cell line (Fig. 3E). Interestingly, loss of CDCP1 in SCaBER cells reduced pAKT, pMEK and pERK1/2 levels (Fig. 3E and supplementary Fig. 2B). Functional analyses showed that CDCP1 depletion reduced both 2D and 3D proliferation (Fig. 3F,G) and migration in this cell line (Fig. 3H). Interestingly, T24 and TCCSUP cells, which are considered non-type^34, 35^, showed a similar behavior to SCaBER when knocked out for CDCP1, with the exception for the 2D proliferation (Supplementary Fig. 2C–F). Collectively, these results demonstrate that CDCP1 expression promotes UC proliferation, while its downregulation reduces proliferation and migration of UC cell lines.

## Discussion

Despite the preponderance of studies identifying CDCP1 as a key contributor to oncogenic events in several cancers^6^, the functional role of CDCP1 and its clinical relevance in UC remains poorly characterized. Only two previous studies from the same laboratory indicate its involvement in BCa^28, 31^. Yang et al. showed that N6-methyladenosine modified the mRNA levels of CDCP1 in response to chemical carcinogens, which promoted CDCP1 translation. The same group also showed that CDCP1 is moderately or highly expressed in most of the BCa samples (n = 33) when compared to para-tumor controls and correlated CDCP1 expression with BCa progression^31^. In line with these results, the first part of our study demonstrated that CDCP1 is overexpressed in UC when compared to NAT and is associated with MIBC. Moreover, the impact of elevated CDCP1 expression in UC was investigated by Kaplan–Meier analysis, which indicated that patients expressing high levels of CDCP1 have significantly poorer OS (Fig. 1D).

In the second part of this study, we further explored the clinical relevance of CDCP1 in UC. Indeed, our results obtained from three different data sets, showed significant enrichment of CDCP1 expression in the Ba/Sq subtype (Fig. 2B,C,E,H). Since CDCP1 is reported to interact with the epidermal growth factor receptor (EGFR)^36^, whose activity is also associated with Ba/Sq tumors in BCa^37, 38^, and a cross-talk between these two proteins is already described in other tumor types^39–41^, further studies investigating the potential CDCP1/EGFR cross-talk are needed. Such studies may show that a combinational therapy targeting CDCP1 and EGFR may be effective for the treatment of Ba/Sq bladder tumors.

Another important clinical aspect presented by our study is the association of CDCP1 with therapy resistance, which has been reported in breast and ovarian cancers^6, 11, 18^. Indeed, the OS of chemotherapy-treated patients expressing high CDCP1 levels was clearly reduced, supporting the hypothesis that CDCP1 is involved in resistance to first-line chemotherapy in UC.

To determine the oncogenic role of CDCP1 in UC, we took advantage of the previously generated mouse model for CDCP1 overexpression in a Cre-dependent manner^12^. Remarkably, CDCP1 overexpression in bladder organoids obtained from this mouse model resulted in larger and well-defined organoids compared to the control groups (Fig. 3B,C). These preliminary results suggest that CDCP1 overexpression may support oncogenesis in bladder urothelium. Additionally, these results encourage the development of a bladder-specific CDCP1-overexpressing transgenic mouse model to further analyze the oncogenic potential of CDCP1 in UC.

At the molecular level, we showed that CDCP1 depletion reduced MEK, ERK and AKT phosphorylation in western blot analyses on SCaBER cells knocked out for CDCP1. The reliance of MAPK/ERK and AKT pathways activation on CDCP1 expression in SCaBER suggests that this protein may play a crucial role in UC progression. Indeed, it was previously reported that the activation of MAPK/ERK and AKT pathways increased tumor growth and cancer cells motility^8, 12^. Functional analysis performed with SCaBER cells showed that CDCP1 depletion reduced their 2D and 3D proliferation and migration (Fig. 3F,G,H). Altogether, these findings demonstrate that the growth and migration abilities of SCaBER cells rely on CDCP1 expression, suggesting that Ba/Sq UC patients could benefit from CDCP1-targeting therapies. Therefore, further studies validating the efficacy of CDCP1 inhibition are clearly encouraged.

## Materials and methods

### Patient cohort and case report

The study was approved by the ethical review board of the Medical Faculty of the University of Bonn (approval number: 036/08 and 093/12) and the Friedrich-Alexander-University Erlangen-Nürnberg (approval number: 329_16B and 97_18Bc). The study was performed in accordance with the Declaration of Helsinki. The study participants were anonymized before their specimens were included in the study cohort. Informed consent was obtained for all the participants. In the first TMA, CDCP1 expression was assessed from patient samples obtained via radical cystectomy or transurethral resection and provided by the University Hospital of Bonn, including benign bladder urothelium and BCa with different stages of disease (T1-T4) as previously described (Table 1)^29, 30^. In the second TMA, CDCP1 expression was assessed in a well-characterized prospective homogenous MIBC cohort treated by radical cystectomy in conjunction with bilateral lymphadenectomy in curative intent at the Department of Urology of the University Hospital of Erlangen^32^.

### Immunohistochemistry

IHC of CDCP1 protein was performed on a VENTANA BenchMark ULTRA autostainer (Ventana) according to an accredited staining protocol in a routine immunohistochemistry facility. A polyclonal anti-CDCP1 primary antibody (#4115, rabbit polyclonal, Cell Signaling, Danvers, MA, US, dilution: 1:75) was used in this study. This antibody was previously used and validated in several other studies^11, 12, 17, 21^. CDCP1 staining was evaluated by two experienced pathologists (OH and GK) and only specific membrane expression of CDCP1 was assessed. Staining intensity was classified as negative (0), weak (1), moderate (2) and strong (3). Negative and weak specimens were considered CDCP1-low, whereas moderate and strong staining were considered CDCP1-high.

### TCGA data analysis

Log2-transformed RSEM (RNA-Seq by Expectation Maximization) RNA sequencing data (RNA-Seq v2) of CDCP1 generated by The Cancer Genome Atlas Research Network (TCGA, http://cancergenome.nih.gov/) were downloaded from the UCSC Xena browser (http://xena.ucsc.edu) for n = 408 UC.

### scRNAseq analysis

Data was downloaded as from Chen et al., 2020^33^, using their interactive Shiny R interface. Analysis and dataset processing was performed using Seurat version 4.1.1 running on a mac OS version 12.2.1 (Monterey). Analysis was performed using standard Seurat dataset processing pipeline. In brief, data was subsetted to include only cells annotated as “epithelial”. Data was visualised using the Nebulosa (version 1.4.0) and scCustomize (version 0.7.0) packages (Table 2). The colour-blind friendly, perceptually uniform and ordered “batlow” colour pallet was used via the R package scico (version 1.3.0). The figure was layed out using Adobe Illustrator version 24.1. Data availability: Publically available scRNAseq data was obtained from chen et al., 2020. Code availability: Code to reproduce scRNAseq data can be found at https://github.com/Eomesodermin.

### Cell culture and functional assays

The human UC cell lines used were purchased from ATCC (ATCC, Manassas, VA, US). Cells were cultured in RPMI1640 (Thermo Fisher Scientific) supplemented with 10% FCS (Thermo Fisher Scientific), 1% streptomycin/penicillin (10.000 units/ml Penicillin and 10.000 µg/ml Streptomycin; Thermo Fisher Scientific) and 1% l-glutamine (200 mM; Thermo Fisher Scientific). For the spheroids formation assay, cells were grown in 1.2% Methylcellulose (Sigma-Aldrich) at a density of 25 × 10^3^ cells/mL as hanging drops and incubated under standard culture conditions for 72 h. The diameter of the formed spheroids was measured with Image J and spheroids volume was calculated with the formula V = 4/3 × π × r^3^. For the migration assay we put 20 × 10^3^ cells on 24-well plate inserts and incubate for 12 h. We then fixed the cells with PFA 4%, performed crystal violet assay and swapped the internal part of the insert´s membrane to eliminate cells which didn´t migrate. We finally counted the migrated cells on the external part of the membrane. For the establishment of the ex vivo organoids model, single cells from the bladder of male CDCP1^pcLSL/+^ mice were isolated as previously described^33^. Single cells were suspended (10^5^ cells/mL) in DMEM, 10% FBS, 100 U/mL penicillin, and 100 µg/mL streptomycin and infected with adenoviruses (rAAV2/1-CMV-GFP and rAAV2/1-CBA-Cre) via spinoculation at 600*g, 1 h, 32 °C. Cells were then incubated for 1 h at 37 °C and 5% CO_2_. 10^6^ cells were suspended in 1 mL of Matrigel (Corning, New York, United States) and 40 µL drops were formed in pre-warmed flat bottom 24-well plates. Matrigel drops were left 30 min to solidify at 37 °C and 5% CO_2_ inverting the 24-well plates and 500 µL of organoids media was added. Organoids were grown for 14 days and pictures were taken. Organoids’ growth was defined by measuring height and length with Image J and calculating the approximate surface. Organoids were then collected and fixed in 4% PFA. For the crystal violet proliferation assay, 2,000 cells were seeded per well in a 96-well plate. After overnight incubation, cells were treated and incubated for three to seven days depending on the experiment. All conditions were run in triplicate wells. For staining, cells were fixed with 37% paraformaldehyde for 10 min, then washed with distilled water and stained 0.05% crystal violet for 30 min. Cells were washed twice with distilled water and dried. 0.1% acetic acid was added per well to solubilize the dye. Finally, the absorbance was measured at a wavelength of 570 nm. The mean values of the triplicate wells were divided by a day zero control. Relative optical density (O.D) was normalized respect to the vehicle control.

### Westernblot and IHC antibodies

For western blot the following antibodies were used: CDCP1 (#4115, Cell Signaling, Danvers, MA, US), pMEK (#9154S, Cell signaling), MEK (#4694S, Cell signaling), pAKT (#9271 T, Cell signaling), AKT (#2920S, Cell Signaling) pERK (#9102S, Cell Signaling), ERK (#4377, Cell Signaling), GAPDH (#2118, Cell Signaling), β-actin (#A2228, Sigma-Aldrich, St. Louis, MO, US). Some of the membranes used for the western blot were cut prior to hybridization. For IHC analysis, the following antibodies were used: CDCP1 (#4115, rabbit polyclonal, Cell Signaling, 1:50), Ki67 (#MSK018, Zytomed, Berlin, Germany, 1:50), p-Erk1/2 (clone 197G2, rabbit monoclonal, Cell Signaling, 1:50), CK5 (clone XM26, mouse monoclonal, Diagnostic BioSystems^®^, USA, dilution 1:50), CK14 (clone SP53, rabbit monoclonal, Cell Marque™, USA, dilution 1:40).

### Quantitative reverse transcriptase-polymerase chain reaction (qRT-PCR)

RNA was isolated with TRIzol method (Invitrogen, Thermo Fisher Scientific, Waltham, MA USA). qRT-PCR was performed using TB Green^®^ Premix Ex Taq™ II (#RR82WR, TaKara, Kusatsu, Japan) on a Quant Studio 5 (applied biosystems, Thermo Fisher Scientific, Wilmington, USA). The primer sequences used were as follow: CDCP1 (Invitrogen Thermo Fisher, forward *TGGTTCCACCCCAGAAATGT*, reverse *GATGATGCACAGACGTTTTAT*), GAPDH (Invitrogen Thermo Fisher Scientific, forward *CTCTGCTCCTCCTGTTCGAC*, reverse *ACGACCAAATCCGTTGACTC*).

### CRISPR-CAS9

A functional sgRNA was generated digesting pSpCas9(BB)-2A-Puro (PX459) (#48139, Addgene, Watertown, MA, US) with BbsI-HF (NEB, Ipswich, MA, US) and ligating a double-stranded DNA oligonucleotide which targets the third exon of CDCP1 with T4 DNA ligase (NEB, Ipswich, MA, US). The double-stranded oligonucleotide was obtained by annealing the following single-stranded oligonucleotides: hCDCP1_KO_BS, 5′-AAACccgtggtcaggatcggaac-3′; hCDCP1_KO_TS, 5′-CACCgttccgatcctgaccacgg-3′. After the ligation, px459-CDCP1^−/–^sgRNA plasmid was transfected in the cells using Lipofectamine™ 3000 (Invitrogen, Thermo Fisher Scientific). After 2 days, the transfected clones were selected with a 4-days Puromycin treatment (0.6 μg/mL) and expanded with a polyclonal approach to obtain stable cell lines.

## Supplementary Information

Supplementary Figures.

Supplementary Information.

## Data Availability

The datasets generated and/or analyzed during the current study are available from the corresponding author on reasonable request.
