# Author Correction: CDCP1 expression is frequently increased in aggressive urothelial carcinoma and promotes urothelial tumor progression

**DOI:** 10.1038/s41598-023-28942-0

**Published:** 2023-02-17

**Authors:** Miriam Saponaro, Sina Flottmann, Markus Eckstein, Oliver Hommerding, Niklas Klümper, Dillon Corvino, Sana Hosni, Anja Schmidt, Nicolas Mönig, Doris Schmidt, Jörg Ellinger, Marieta Toma, Glen Kristiansen, Tobias Bald, Andrea Alimonti, Manuel Ritter, Michael Hölzel, Abdullah Alajati

**Affiliations:** 1 Department of Urology and Pediatric Urology, University Medical Center Bonn (UKB), Venusberg-Campus 1, 53127 Bonn, Germany; 2 Institute of Pathology, University Hospital Erlangen, Friedrich-Alexander-Universität Erlangen-Nürnberg (FAU), Erlangen, Germany; 3 Institute of Pathology, University Medical Center Bonn (UKB), Bonn, Germany; 4 Institute of Experimental Oncology, University Medical Center Bonn (UKB), Bonn, Germany; 5 Institute of Oncology Research, Università Della Svizzera Italiana, Bellinzona, Switzerland

Correction to: *Scientific Reports* https://doi.org/10.1038/s41598-022-26579-z, published online 02 January 2023

Tables 1 and 2 in the original version of this Article presented redundant for the research information. To increase their readability, data in both Tables was summarized.

The original Article has been corrected.

