# Supplementary material for: CDCP1 expression is frequently increased in aggressive urothelial carcinoma and promotes urothelial tumor progression
Source: Sci Rep. 2023 Jan 2;13:73. doi: 10.1038/s41598-022-26579-z (PMC9807563; doi:10.1038/s41598-022-26579-z)
Supplement: Supplementary file 1 — Supplementary Figures. [file 41598_2022_26579_MOESM1_ESM.pdf]

Supplementary Figure 1

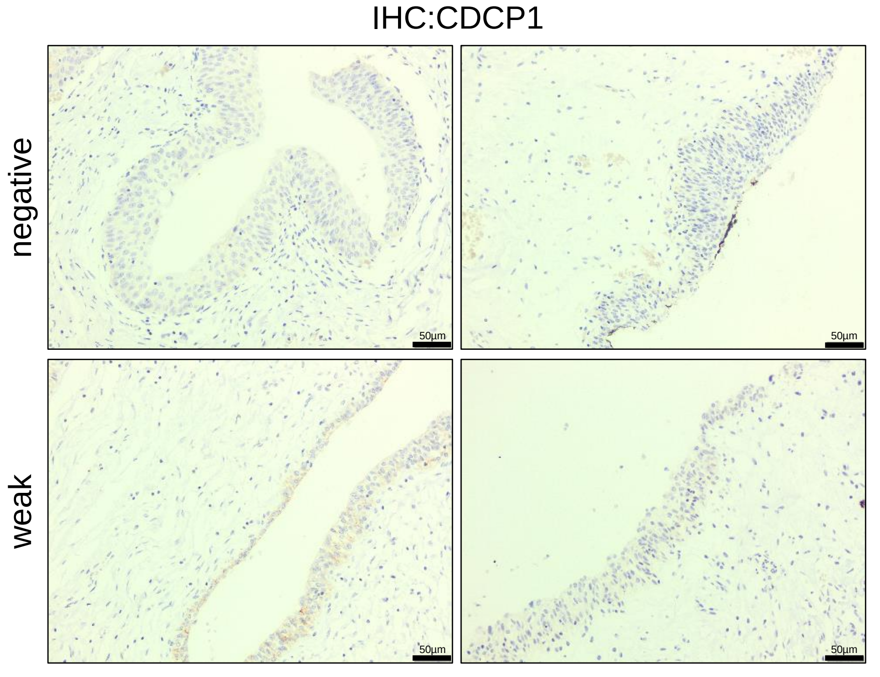

Supplementary Figure 1. Representative images of IHC for CDCP1 in normal urothelium or normal adjacent tissue showing negative to weak staining intensity of CDCP1. Scale Bars 50µm.

Supplementary Figure 2

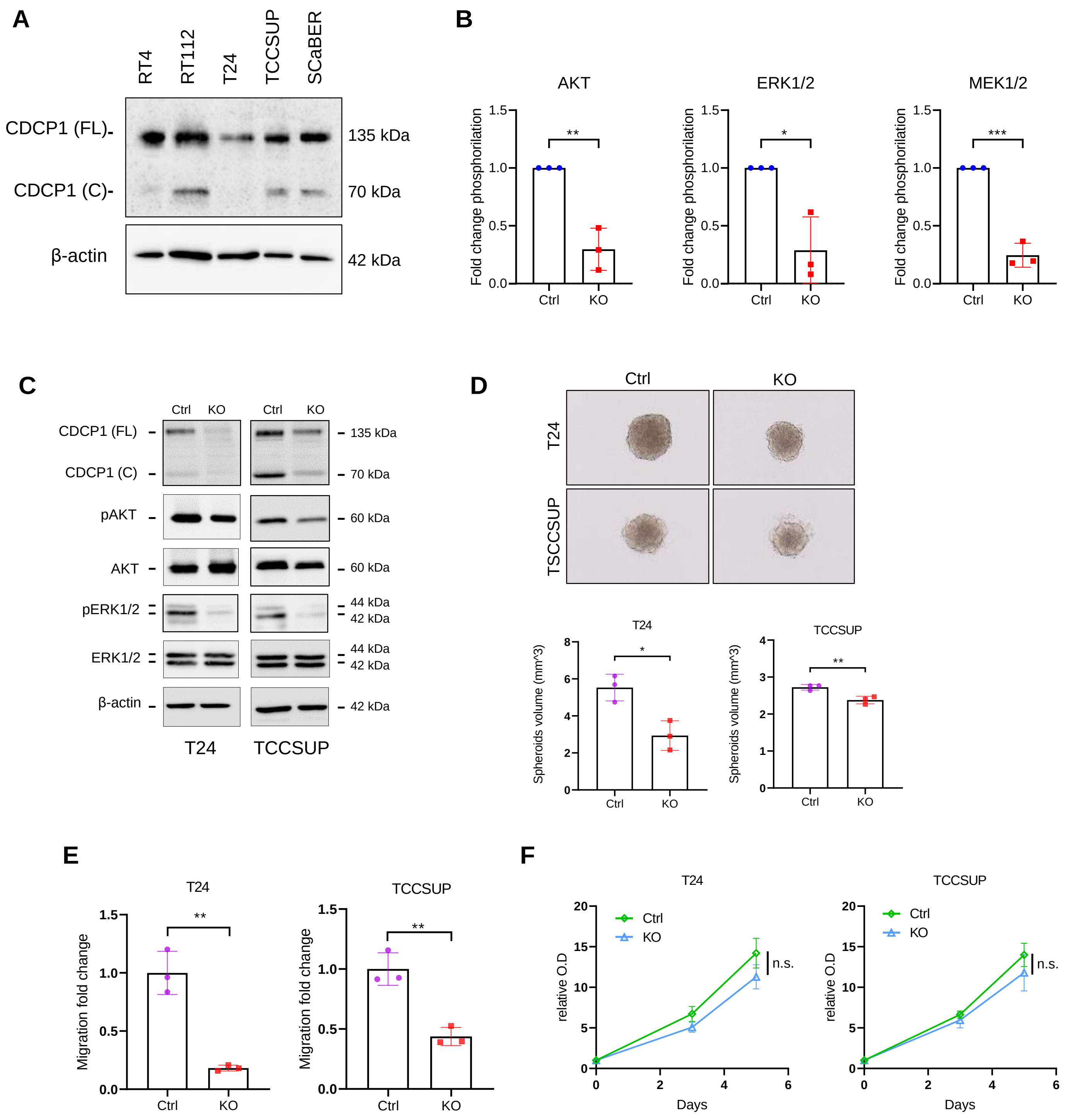

Supplementary Figure 2. (A) Western blot analysis of CDCP1 in different BCa cell lines. (FL) indicates the full length of CDCP1 and (C) indicates the cleaved form of CDCP1. β-actin expression was used as loading control. (B) Bar graphs represent the fold change of normalized pAKT, pErk1/2 and pMEK1/2 to the total protein level in SCaBER cell lines. Error bars indicate SD. *P < 0.05, **P < 0.01, ***P < 0.001. (C) Western blot analysis of CDCP1 and major downstream targets of CDCP1 signaling in CDCP1 expressing UC cell lines and their CDCP1-/- counterparts. (D) Representative images of the spheroids originated from the CDCP1+ (Ctrl) and CDCP1-/- (KO) UC cell lines. Bar graphs show the quantification of the spheres volume. Error bars indicate SD. *P < 0.05, **P < 0.01. Statistical test: two-tailed unpaired t test. (E) Migration fold change of the CDCP1 expressing cells (Ctrl) compared to the CDCP1-/- (KO). Error bars indicate SD. **P < 0.01. Statistical test: two-tailed unpaired t test. (F) Relative O.D (proliferation) change of the CDCP1 expressing UC cell lines (Ctrl) compared to the CDCP1-/- (KO). Error bars indicate SD. ***P < 0.001. Statistical test: two-tailed unpaired t test.
