# Supplementary material for: CDCP1 expression is frequently increased in aggressive urothelial carcinoma and promotes urothelial tumor progression
Source: Sci Rep. 2023 Jan 2;13:73. doi: 10.1038/s41598-022-26579-z (PMC9807563; doi:10.1038/s41598-022-26579-z)
Supplement: Supplementary file 2 — Supplementary Information. [file 41598_2022_26579_MOESM2_ESM.pdf]

Figure 1: Full-length, original, unprocessed blots to confirm specific detection of the target antigen

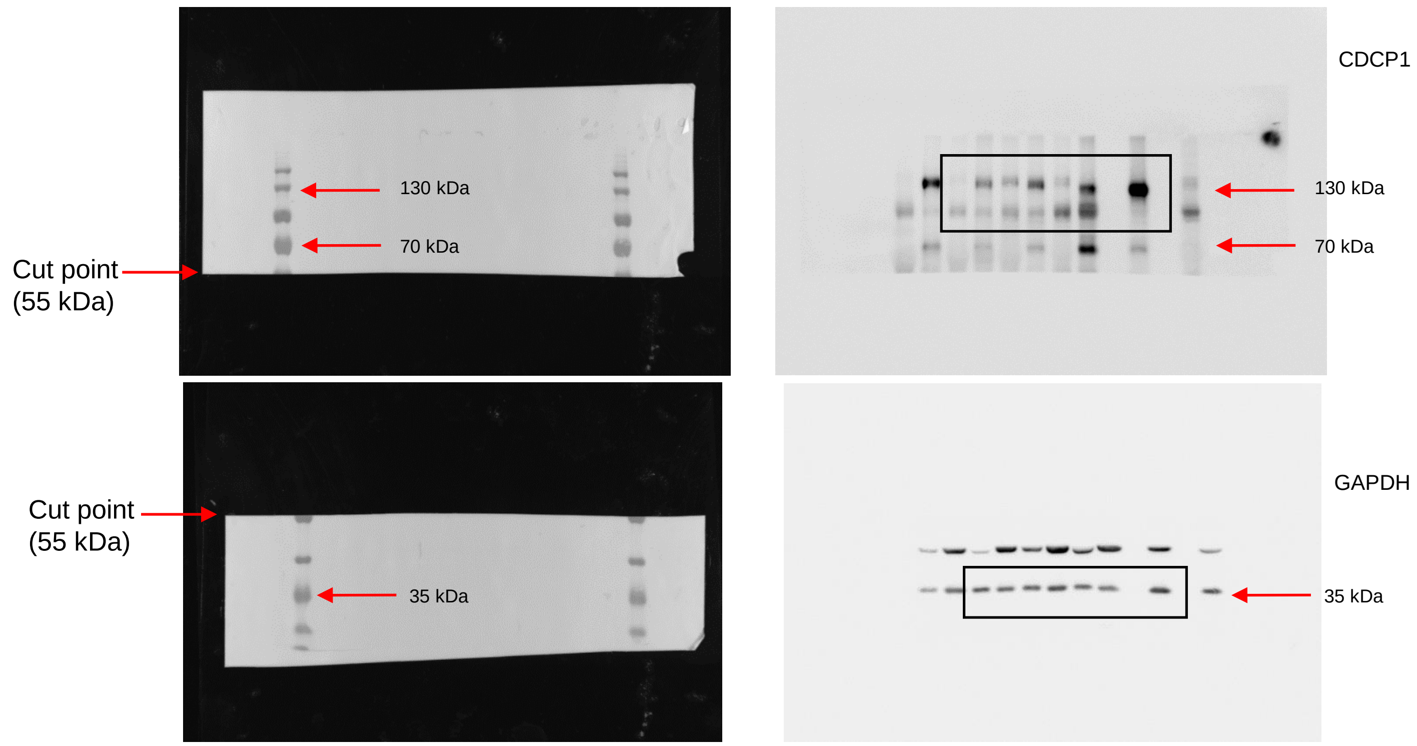

Figure 2: Full-length, original, unprocessed blots to confirm specific detection of the target antigen

CDCP1

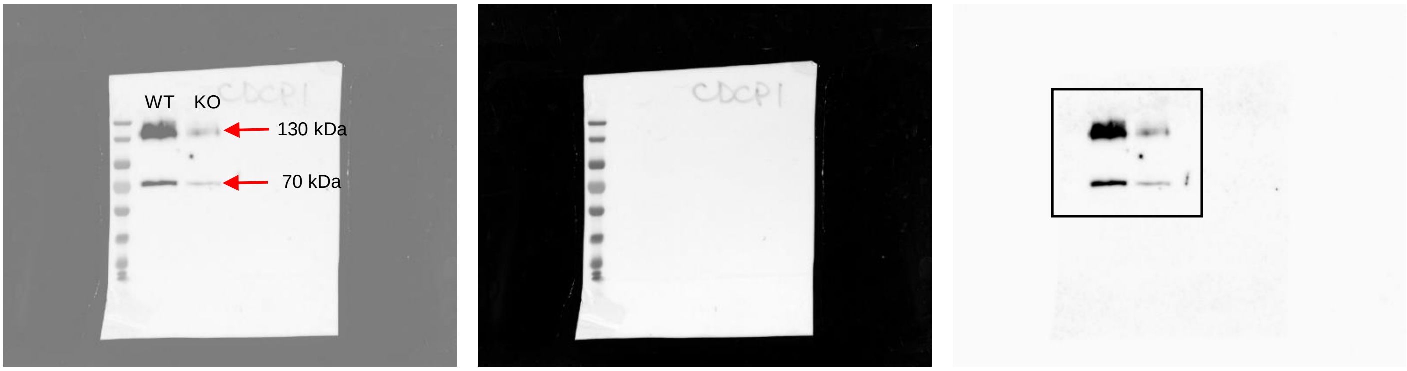

Phospho AKT

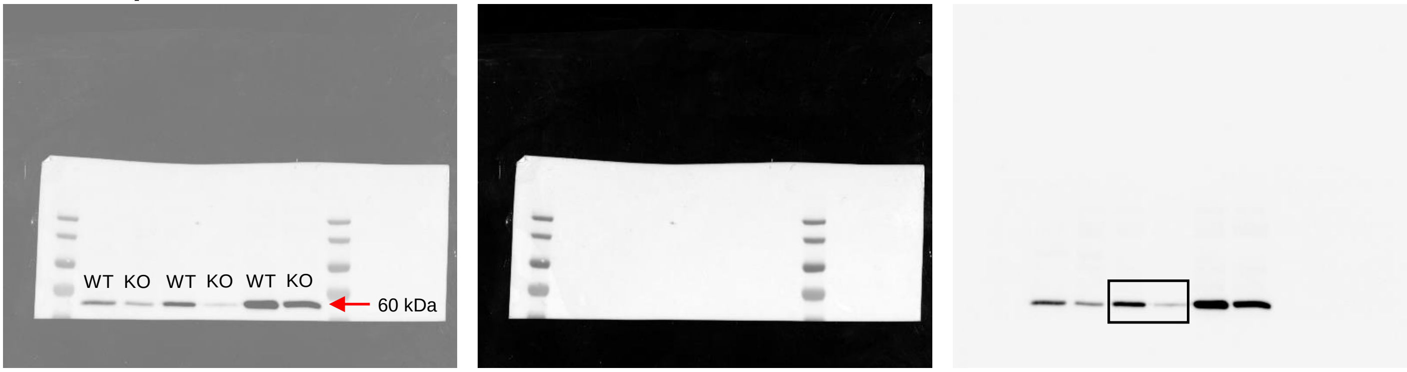

Pan AKT

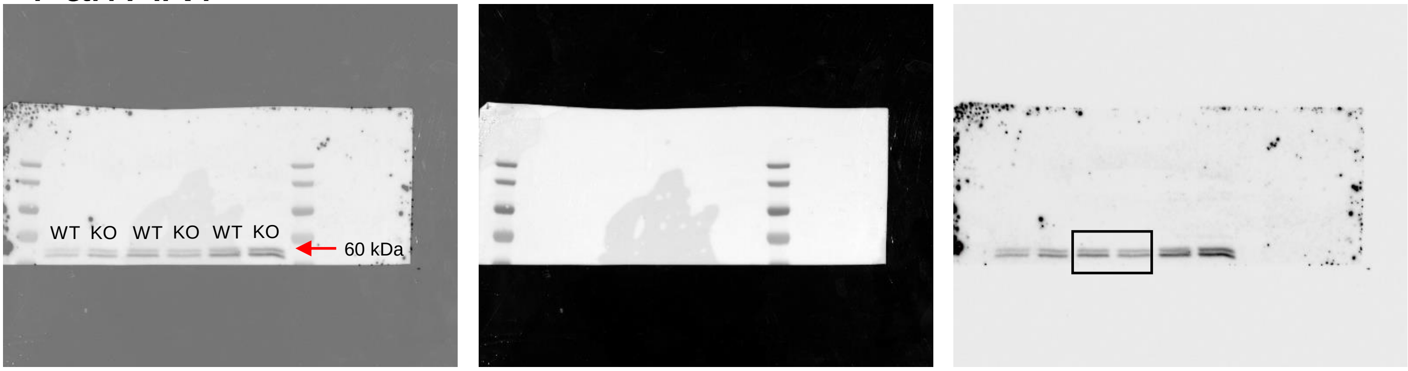

Figure 2: Full-length, original, unprocessed blots to confirm specific detection of the target antigen

Phospho MEK

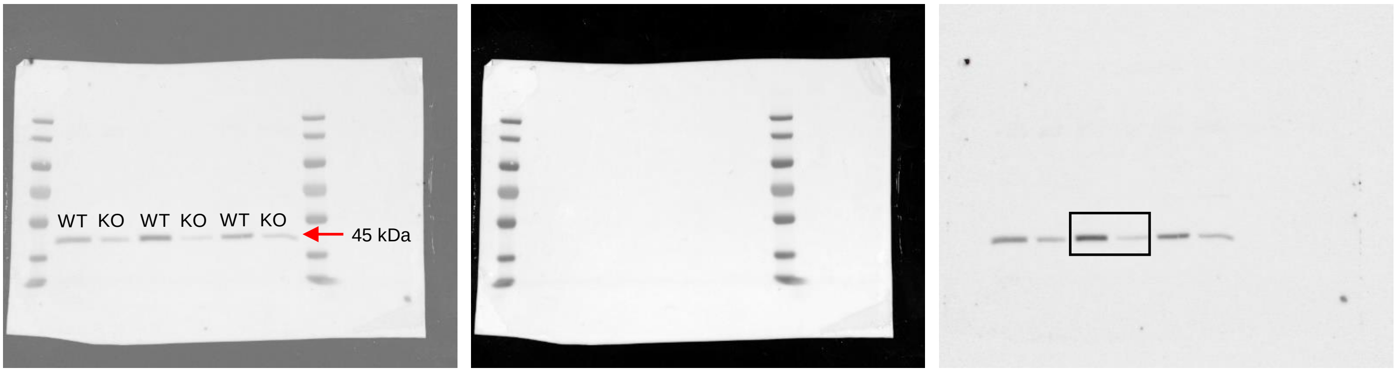

MEK

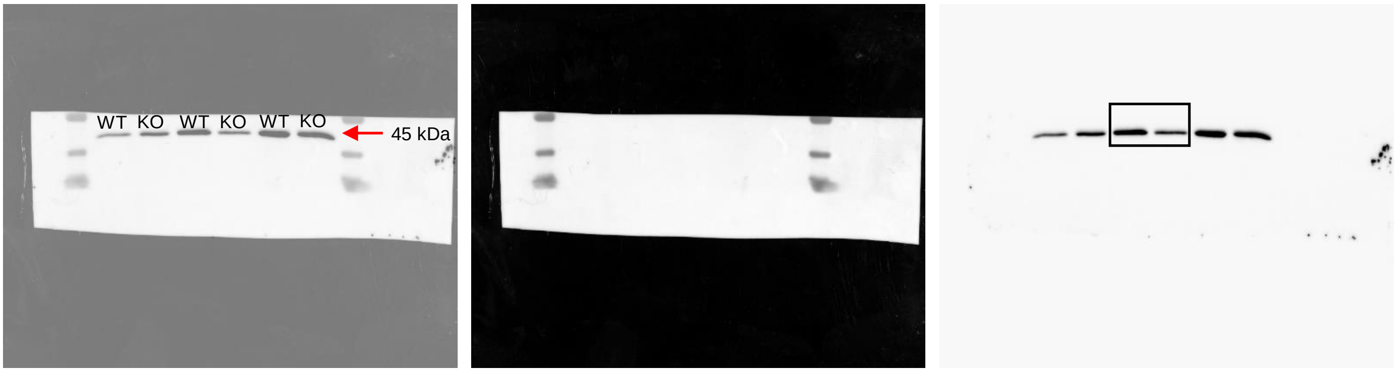

Phospho ERK

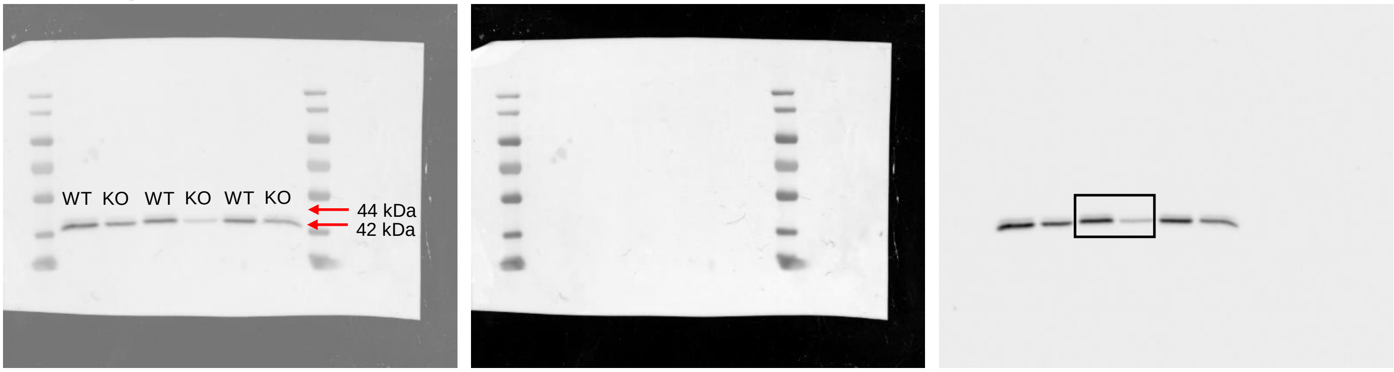

ERK

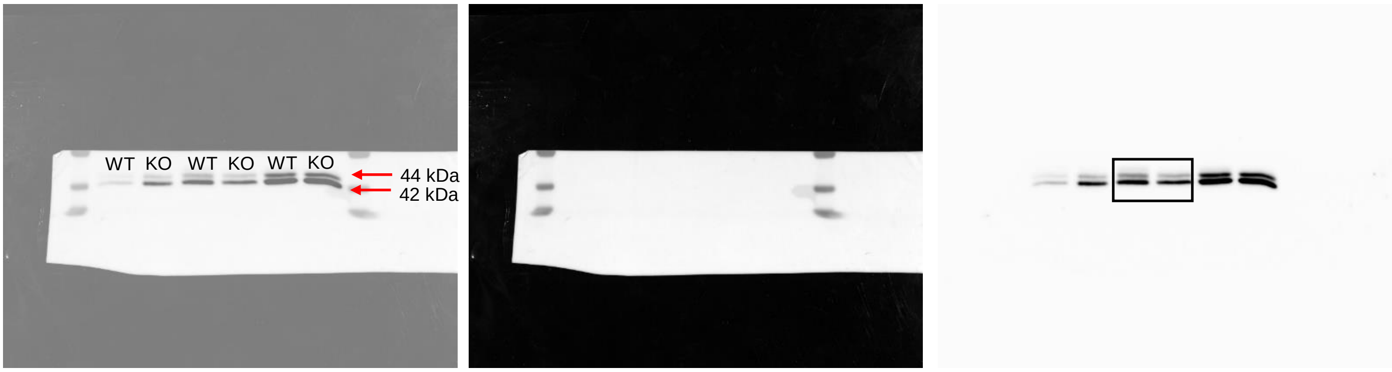

Figure 2: Full-length, original, unprocessed blots to confirm specific detection of the target antigen

β-actin

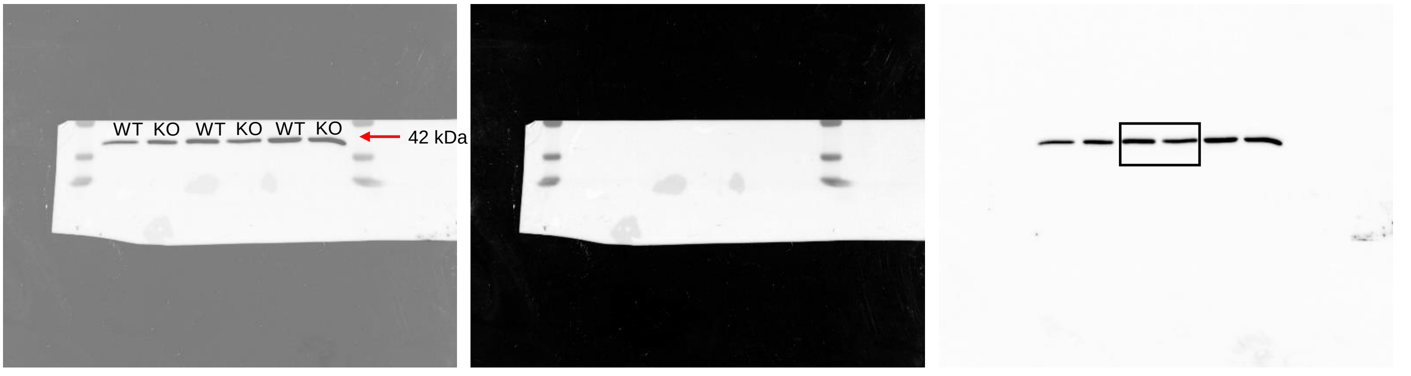

Supplementary Figure2: Full-length, original, unprocessed blots to confirm specific detection of the target antigen

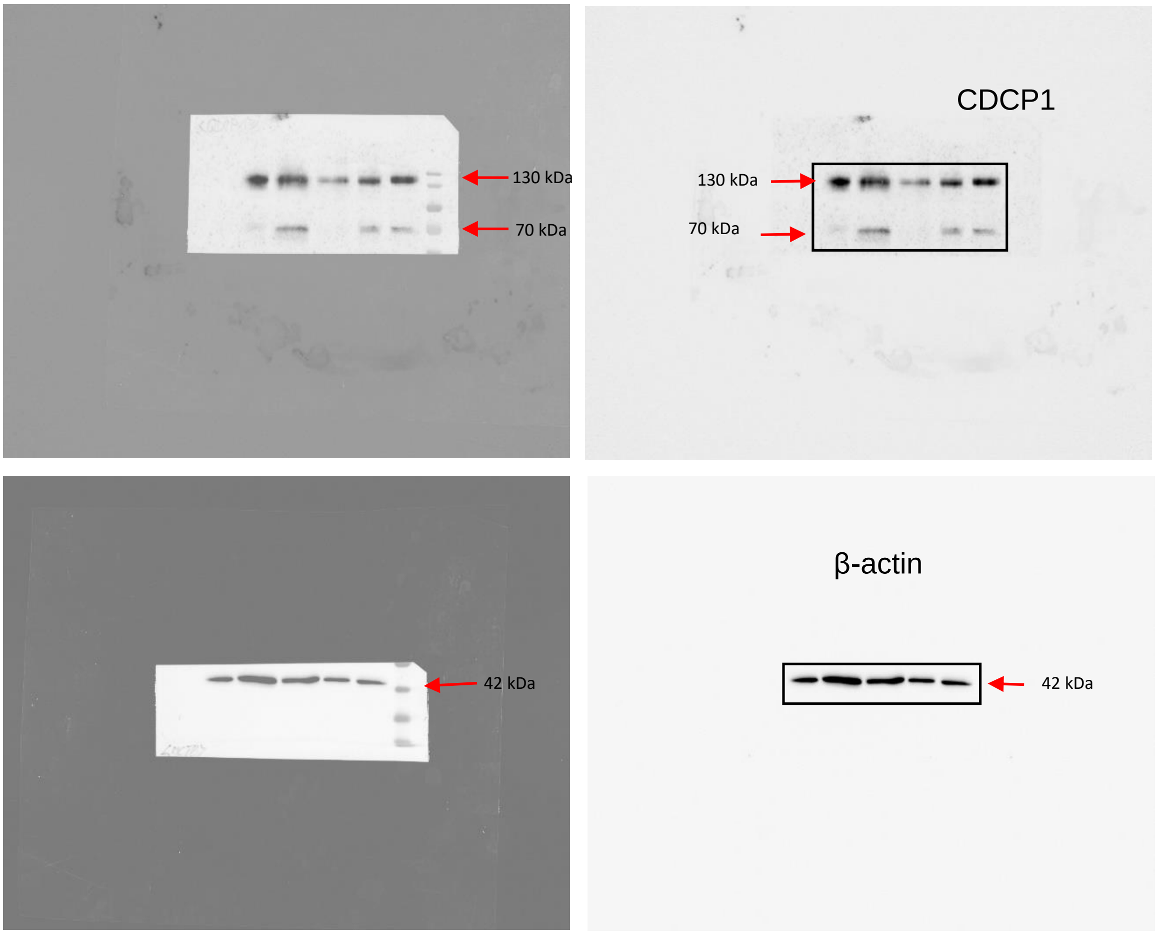

Supplementary Figure2: Full-length, original, unprocessed blots to confirm specific detection of the target antigen

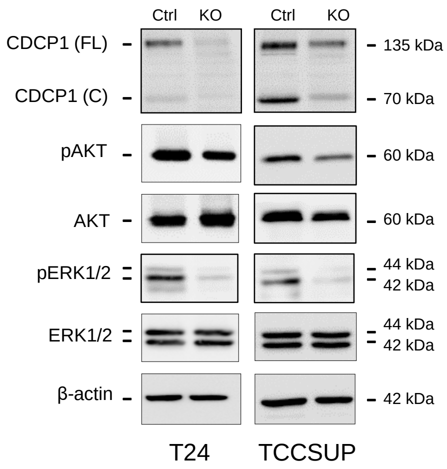

T24

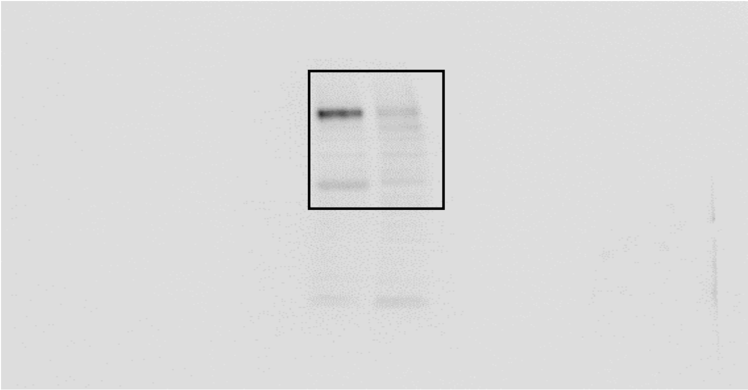

CDCP1

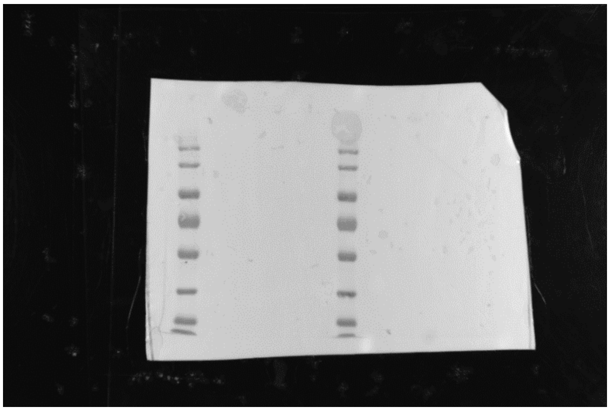

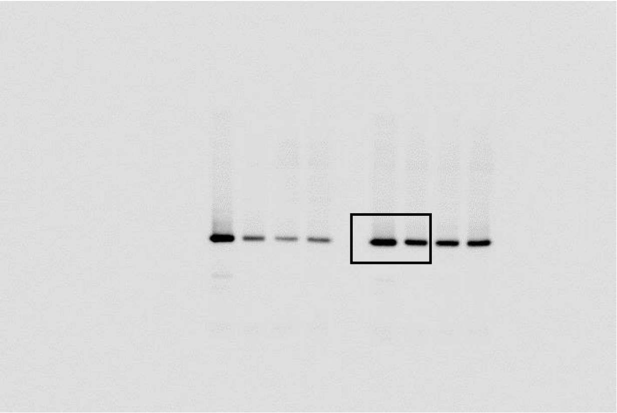

pAKT

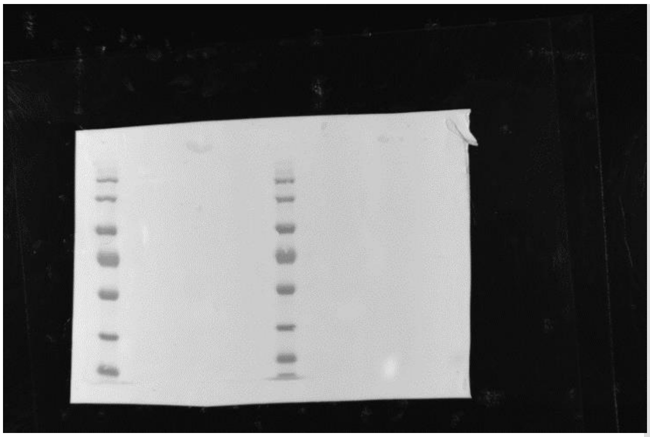

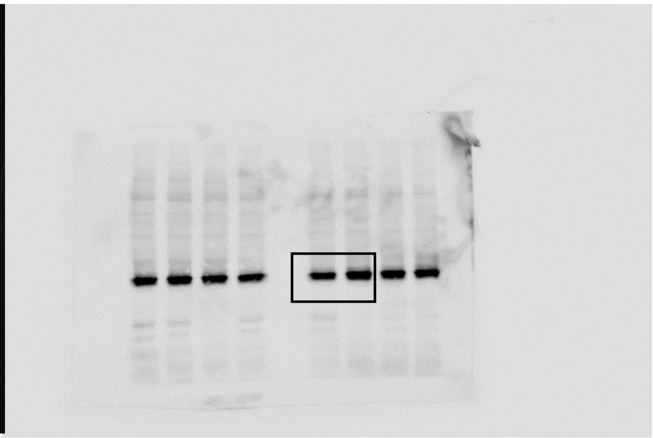

AKT

Supplementary Figure2

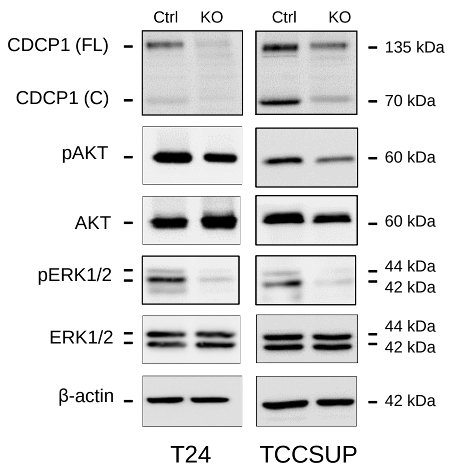

TCCSUP

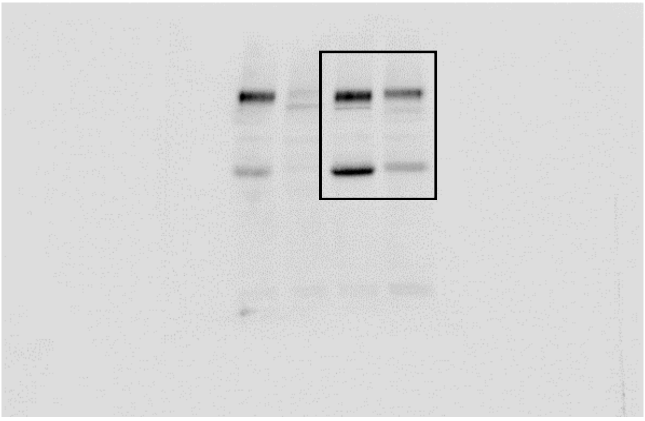

CDCP1

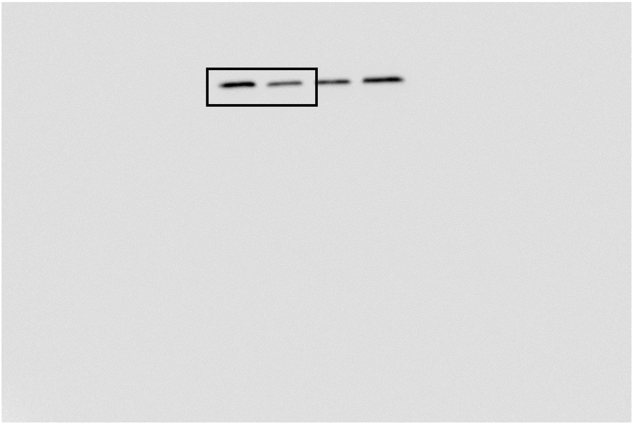

pAKT

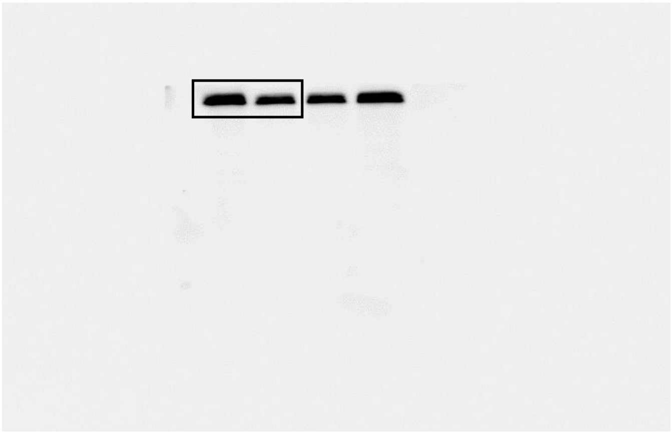

AKT

Supplementary Figure2

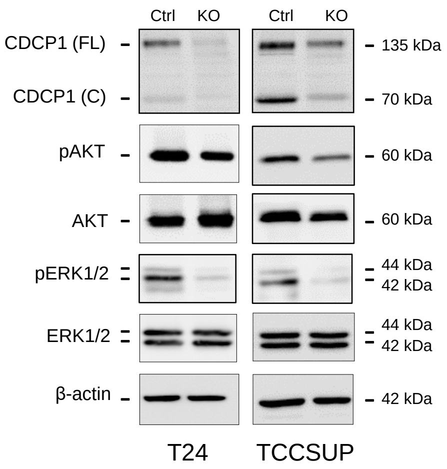

T24

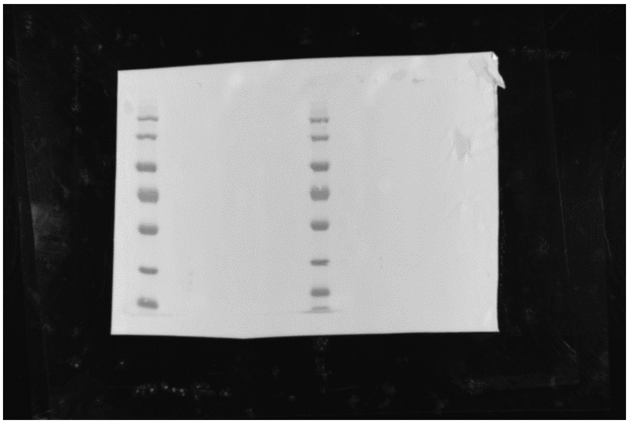

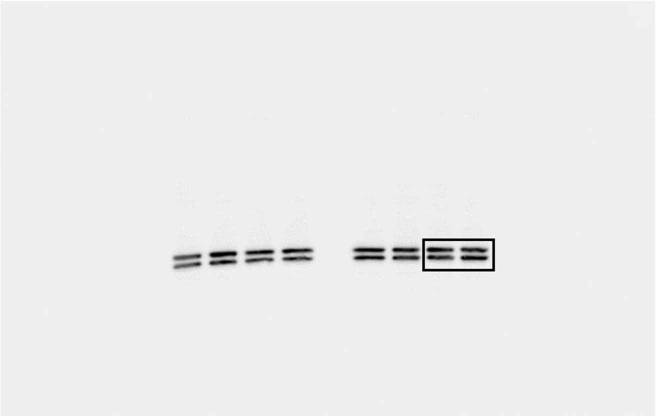

ERK1/2

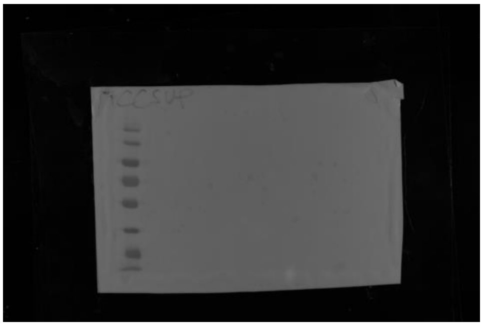

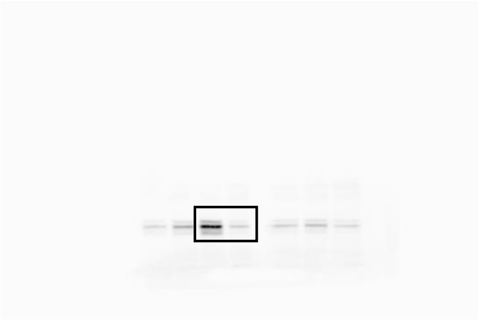

pERK1/2

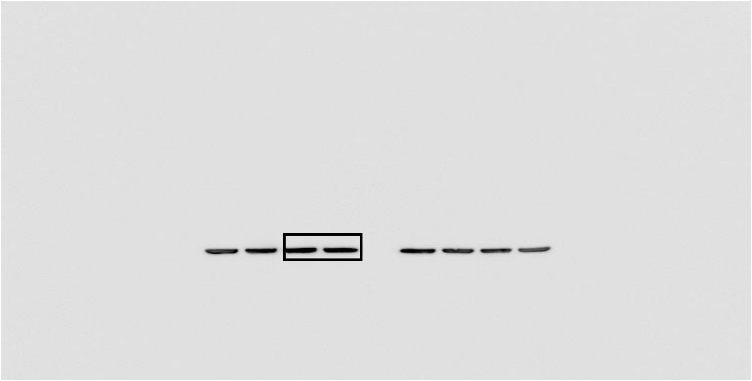

β-ACTIN

Supplementary Figure2

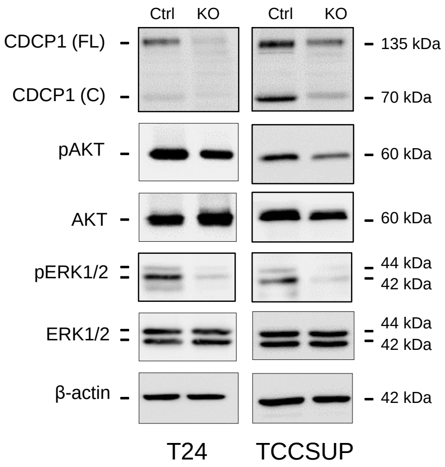

TCCSUP

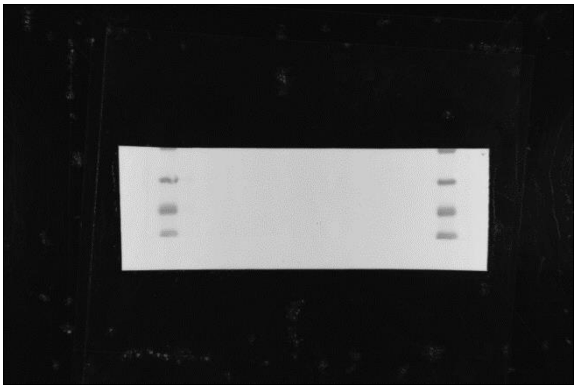

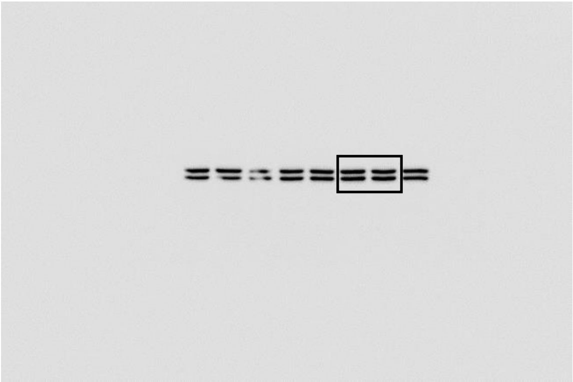

ERK1/2

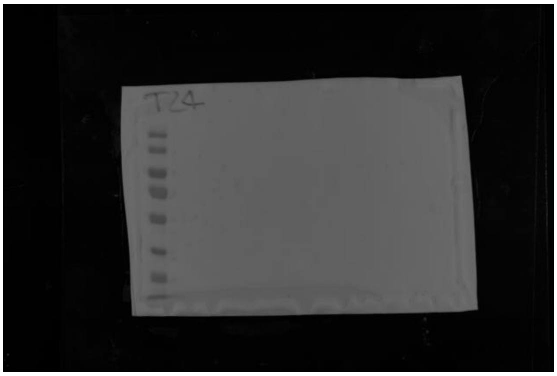

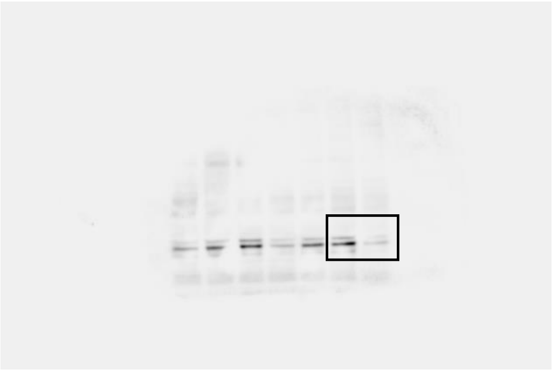

pERK1/2

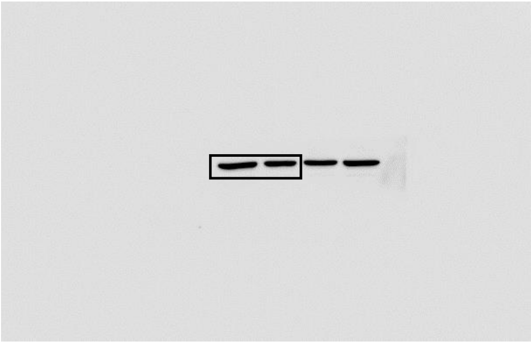

β-ACTIN
